# Supplementary material for: Bacterial capsular polysaccharides with antibiofilm activity share common biophysical and electrokinetic properties
Source: Nat Commun. 2023 May 3;14:2553. doi: 10.1038/s41467-023-37925-8 (PMC10156666; doi:10.1038/s41467-023-37925-8)
Supplement: Supplementary file 1 — Supplementary Information [file 41467_2023_37925_MOESM1_ESM.pdf]

SUPPLEMENTARY INFORMATION

Bacterial capsular polysaccharides with antibiofilm activity share common biophysical and electrokinetic properties

Joaquín Bernal-Bayard *et al.*

This PDF file includes:

Supplementary Tables 1 to 3

Supplementary Figures 1 to 8

Supplementary References

SUPPLEMENTARY TABLES

Supplementary Table 1. Name, origin, primary structure and properties of the polysaccharides used in this study[1].

Name[1]	Bacteria	Primary structure	Antibiofilm activity	References
PnPS1	*Streptococcus pneumoniae* serotype 1	2/3-(OAc)$_{68\%}$<br>I<br>[3)-α-AAT*p*-(1➔4)-α-D-Gal*p*A-(1➔3)-α-D-Gal*p*A-(1➔]$_n$	-	1 2
PnPS2	*Streptococcus pneumoniae* serotype 2	[4)-β-D-Glc*p*-(1➔3)-α-L-Rha*p*-(1➔3)-α-L-Rha*p*-(1➔3)-β-L-Rha*p*-(1➔]$_n$<br>2<br>↑<br>1<br>α-D-GlcA*p* -(1➔6)- α-D-Glc*p*	-	3
**PnPS3**	*Streptococcus pneumoniae* serotype 3	[4)-β-D-Glc*p*-(1➔3)-β-D-Glc*p*A-(1➔]$_n$	**G+ / ~ G-**	4 2
PnPS4	*Streptococcus pneumoniae* serotype 4	[3)-β-D-Man*p*NAc-(1➔3)-α-L-Fuc*p*NAc-(1➔3)-α-D-Gal*p*NAc-(1➔4)-α-D-Gal*p*-(2,3-(s)-pyruvate)(1➔]$_n$	-	5 6
PnPS5	*Streptococcus pneumoniae* serotype 5	[4)-β-D-Glc*p*-(1➔4)-α-L-Fuc*p*NAc-(1➔3)-β-D-Sug*p*-(1➔]$_n$<br>3<br>↑<br>1<br>α-L-Pne*p*NAc-(1➔2)-β-D-Glc*p*A	-	7 6
PnPS6B	*Streptococcus pneumoniae* serotype 6B	[2)-α-D-Gal*p*-(1➔3)-α-D-Glc*p*-(1➔3)-α-L-Rha*p*-(1➔4)-D-Ribitol-5-*P*-(O➔]$_n$	-	8 2
PnPS7F	*Streptococcus pneumoniae* serotype 7F	2-OAc<br>I<br>[6)-α-D-Gal*p*-(1➔3)-β-L-Rha*p*-(1➔4)-β-D-Glc*p*-(1➔3)-β-D-Gal*p*NAc-(1➔]$_n$<br>2 ... 4<br>↑ ... ↑<br>1 ... 1<br>β-D-Gal*p* ... α-D-Glc*p*NAc-(1➔ 2)-α-L-Rha*p*	-	9 2
PnPS8	*Streptococcus pneumoniae* serotype 8	[4)-β-D-Glc*p*A-(1➔4)-β-D-Glc*p*-(1➔4)-α-D-Glc*p*-(1➔4)-α-D-Gal*p*-(1➔]$_n$	-	10 2
PnPS9N	*Streptococcus pneumoniae* serotype 9N	[4)-α-D-Glc*p*A-(1➔3)-α-D-Glc*p*-(1➔3)-β-D-Man*p*NAc-(1➔4)- β-D-Glc*p*-(1➔4)-α-D-Glc*pNAc*-(1➔]$_n$	-	11 2
PnPS9V	*Streptococcus pneumoniae* serotype 9V	[4)-α-D-Glc*p*-(1➔4)-α-D-Glc*p*A-(1➔3)-α-L-Gal*p*-(1➔3)-β-D-Man*p*NAc-(1➔4)-β-D-Glc*p*-(1➔]$_n$<br>I ... I<br>2/3-(OAc)$_{80\%}$ ... 4/6-(OAc)$_{113\%}$	-	12 2

PnPS10A	*Streptococcus pneumoniae* serotype10A	β-D-Gal*p*<br>1<br>↓<br>6<br>[5)-β-D-Gal*f*-(1→3)-β-D-Gal*p*-(1→4)-β-D-Gal*pNAc*-(1→3)-α-D-Gal*p*-(1→2)-D-Ribitol-5-*P*-(O→]*n*<br>3<br>↑<br>1<br>β-D-Gal*f*	-	13 2
PnPS11A	*Streptococcus pneumoniae* serotype 11A	2/3-(OAc)$_{110\%}$ 4/6-(OAc)$_{50\%}$<br>I I<br>[6)-α-D-Glc*p*-(1→4)-α-D-Gal*p*-(1→3)-β-D-Gal*p*-(1→4)-β-D-Glc*p*-(1→]*n*<br>4<br>I<br>*O)-P*-1-Glycerol	-	14 2
<u>PnPS12F</u>	*Streptococcus pneumoniae* serotype 12F	[4)-α-L-Fuc*p*NAc-(1→3)-β-D-Gal*p*NAc-(1→4)-β-D-Man*p*NAcA-(1→]*n*<br>3 3<br>↑ ↑<br>1 1<br>α-D-Gal*p* α-D-Glc*p*-(1→2)-α-D-Glc*p*	**G- only**	15 2
PnPS14	*Streptococcus pneumoniae* serotype 14	[6)-β-D-Glc*pN*Ac-(1→3)-β-D-Gal*p*-(1→4)-β-D-Glc*p*-(1→]*n*<br>4<br>↑<br>1<br>β-D-Gal*p*	-	16 2
PnPS15B	*Streptococcus pneumoniae* serotype15B	[6)-β-D-Glc*pN*Ac-(1→3)-β-D-Gal*p*-(1→4)-β-D-Glc*p*-(1→]*n*<br>4<br>↑<br>1<br>α-D-Gal*p*-(1→ 2)- β-D-Gal*p*<br>I 3<br>I<br>2/3/4/6-(OAc)$_{85\%}$ *O)-P*-2-Glycerol	-	17 6
PnPS17F	*Streptococcus pneumoniae* serotype 17F	2-OAc<br>I<br>[3)-β-L-Rha*p*-(1→4)-D-β-D-Glc*p*-(1→3)-α-D-Gal*p*-(1→3)-β-L-Rha*p*-(1→4)-α-L-Rha*p*-(1→2)-D-Ara-ol-1-*P*-(O→]*n*<br>4<br>↑<br>1<br>β-D-Gal*p*	-	18 2
<u>PnPS18C</u>	*Streptococcus pneumoniae* serotype 18C	6-(OAc)$_{30\%}$<br>I<br>α-D-Glc*p*<br>1<br>↓<br>2<br>[4)-β-D-Glc*p*-(1→4)-β-D-Gal*p*-(1→4)-α-D-Glc*p*-(1→3)-α-L-Rha*p*(1→]*n*<br>3<br>I<br>*O)-P*-2-Glycerol	**G+ only**	19 6
PnPS19A	*Streptococcus pneumoniae* serotype 19A	[4)-β-D-Man*p*NAc-(1→4)-α-D-Glc*p*-(1→3)-α-L-Rha*p*-1-*P*-(O→]*n*	-	20 2
PnPS19F	*Streptococcus pneumoniae* serotype 19F	[4)-β-D-Man*p*NAc-(1→4)-α-D-Glc*p*-(1→2)-α-L-Rha*p*-1-*P*-(O→]*n*	-	21 2
PnPS20	*Streptococcus pneumoniae* serotype 20	6-(OAc)$_{90\%}$<br>I<br>[6)-α-D-Glc*p*-(1→6)-β-D-Glc*p*-(1→3)-β-D-Gal*f*-(1→3)-β-D-Glc*p* -(1→3)-α-D-Glc*pN*Ac-1-*P*-(O→]*n*<br>I 4<br>5-(OAc)$_{90\%}$ ↑<br>1<br>β-D-Gal*f*<br>I<br>2-(OAc)$_{90\%}$	-	12 2
PnPS22F	*Streptococcus pneumoniae* serotype 22F	β-D-Glc*p*<br>1<br>↓<br>3<br>[4)-β-D-Glc*p*A-(1→4)-β-L-Rha*p*-(1→4)-α-D-Glc*p*-(1→3)-α-D-Gal*f*-(1→2)-α-L-Rha*p*-(1→]*n*<br>I<br>2-(OAc)$_{80\%}$	-	22 2
PnPS23F	*Streptococcus pneumoniae* serotype 23F	α-L-Rha*p*<br>1<br>↓<br>2<br>[4)-β-D-Glc*p*-(1→4)-β-D-Gal*p*-(1→4)-β-L-Rha*p*-(1→]*n*<br>3<br>I<br>*O)-P*-2-Glycerol	-	23 2
PnPS33F	*Streptococcus pneumoniae* serotype 33F	[3)-β-D-Gal*p*-(1→3)-α-D-Gal*p*-(1→3)-β-D-Gal*f*-(1→3)-β-D-Glc*p* -(1→5)-β-D-Gal*f*(1→]*n*<br>2 I<br>↑ 2-(OAc)$_{50\%}$<br>1<br>α-D-Gal*p*	-	24 2
CWPS	Non-capsulated *Streptococcus pneumoniae* strain CSR SCS2	[6)-β-D-Glc*p*-(1→3)-α-AAT*p*-(1→4)-α-D-Gal*p*NAc-(1→3)-β-D-Gal*p*NAc-(1→1)-D-Ribitol-5-*P*-(O→]*n*<br>6<br>I<br>O)-*P*-Cho	-	25

**Vi**	*Salmonella enterica Serovar Typhi*	3-(OAc)$_{90\%}$ I [4)-α-D-Gal*p*NAcA-(1➔]$_n$	**G+ / ~ G-**	26
**PRP**	*Haemophilus influenzae* serotype b	[3)-β-D-Rib*f*-(1➔1)-D-Ribitol-5-*P*-(O➔]$_n$	**G+ / ~ G-**	27
**MenA**	*Neisseria meningitidis* serogroup A	3/4-(OAc)$_{90\%}$ I [6)-α-D-Man*p*NAc-1-*P*-(O➔]$_n$	**G+ & G-**	28
**MenC**	*Neisseria meningitidis* serogroup C	7/8-(OAc)$_{90\%}$ I [9)-α-D-Neu*p5Ac*-(2➔]$_n$	**G+ & G-**	28
**MenY**	*Neisseria meningitidis* serogroup Y	7/9-(OAc)$_{\sim 50\%}$ I [4)-α-D-Neu*p*5Ac-(2➔6)-α-D-Glc*p*-(1➔]$_n$	**G+ & G-**	28
**MenW135**	*Neisseria meningitidis* serogroup W135	7/9-(OAc)$_{\sim 60\%}$ I [4)-α-D-Neu*p*5Ac-(2➔6)-α-D-Gal*p*-(1➔]$_n$	**G+ & G-**	28
**G2cps**	*Escherichia coli* strain CFT073	3-(OAc)$_{100\%}$ I [4)-α-D-Gal*p*-(1➔2)-Glycerol-3-*P*-(O➔]$_n$	**G+ & G-**	[29], this study

In bold: active broad spectrum antibiofilm polysaccharides. Underlined: active narrow spectrum antibiofilm polysaccharides.

[1]: All polysaccharides produced and purified from *S. pneumoniae*, *N. meningiditis*, *S.* Typhi and *H. influenzae* strains used in this study were obtained from Sanofi, Marcy l'Etoile France and Swiftwater, USA.

Supplementary Table 2. Electrokinetic and size properties of the polysaccharidic macromolecules tested in this work.

**Polysaccharide**	**$\lvert \rho_o/F \rvert$ (mM)**	**$1/\lambda_o$ (nm)**	**Size *d* (nm) ± standard deviation**
**Vi**			15.6 ± 0.7
PnPS4	9.7	3.3	380.2 ± 39.1
PnPS10A	14.8	2.3	25.9 ± 4.3
PnPS15B	15.9	2.4	29.1 ± 2.5
PnPS20	13.2	2.4	32.1 ± 2.5
PnPS19A	15.8	2.9	20.9 ± 4.2
PnPS8	20.5	2.5	23.4 ±1.4
**MenA**			12.1 ± 0.7
**MenC**	52.8	1.7	25.4 ± 1.9
**MenY**	48.4	1.5	26.9 ± 1.8
**PnPS3**	18.7	3.2	26.3 ± 3.9
PnPS1	20.1	2.5	26 ± 2.3
PnPS9V	25.7	1.4	8.7 ± 1.6
**PRP**	20.3	3.4	28.4 ± 5
**G2cps**	18.1	3.2	40±15
PnPS7F	10.9	3.1	39.4 ± 2.7
PnPS22F	15.6	2.3	26.3 ± 1.9
PnPS14	30.3	1.6	15.2 ±5.1
**MenW135**			28.3 ± 3.1
PnPS18C			26.7 ± 2.2
PnPS12F			29.5 ± 3.3

In bold*:* active broad-spectrum antibiofilm polysaccharides. Underlined: active narrow spectrum antibiofilm polysaccharides**.** In black: inactive polysaccharides. *d* stands for the diameter of the macromolecules (*d*=2*b* where *b* is the particle radius involved in eq. 1). The values ρ_o/F and $1/\lambda_o$ for the polysaccharides Vi, MenA, MenW135 and PnPS18C, PnPS12F fall within the space solution defined by the red and blue zones in Figure 6, respectively.

Supplementary Table 3. Bacterial strains used in this study.

Strain	Relevant characteristics	Source/Reference
Strains producing antibiofilm polysaccharides		
E. coli CFT073	Uropathogenic *E.coli* - group 2 capsule producer	30
Strains used as biofilm test panel		
E. coli K-12 MG1655 *F'tet ∆traD*	*traD*::apra plasmid, $Apra^R$, Tet^R	29
Enterobacter cloacae 1092		31
Klebsiella pneumoniae U21	Clinical isolate	31
Staphylococcus aureus 15981		32
Staphylococcus epidermidis 047		33

SUPPLEMENTARY FIGURES

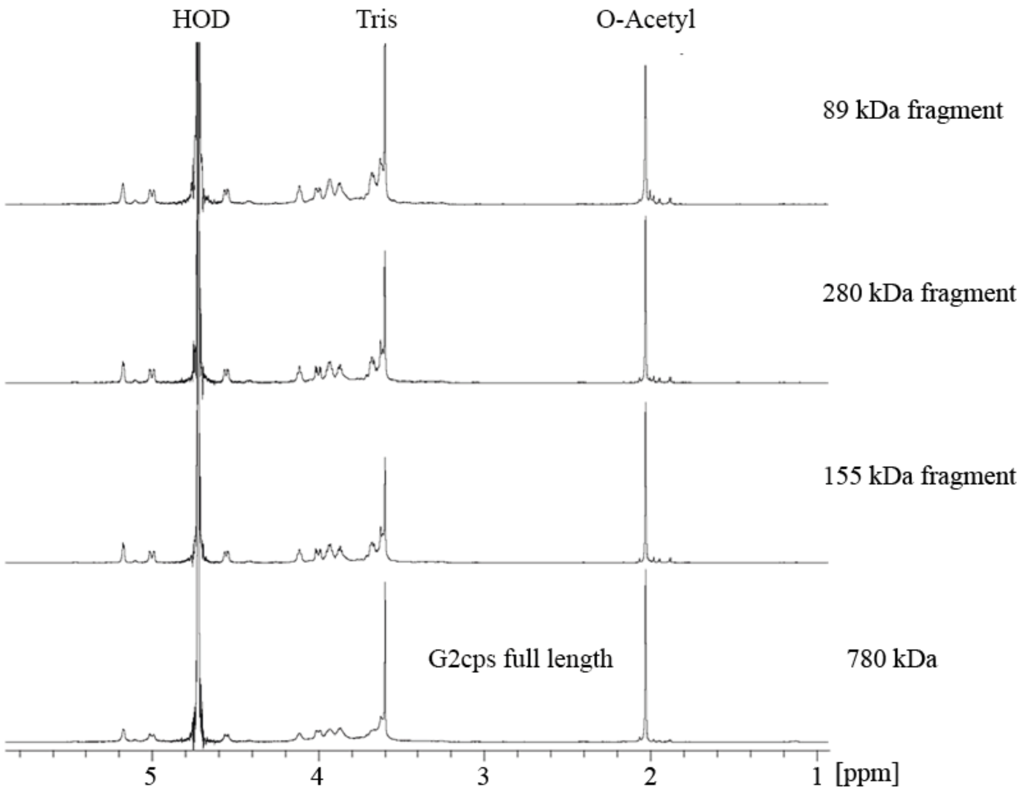

Supplementary Figure 1**. ^{1}H NMR spectra of native G2cps polysaccharide and corresponding fragments obtained by radical oxidation hydrolysis**. The analysis was performed at 20°C. On each spectrum is indicated the molecular weight *Mw* (kDa) determined by HPSEC. HOD: signal of residual water.

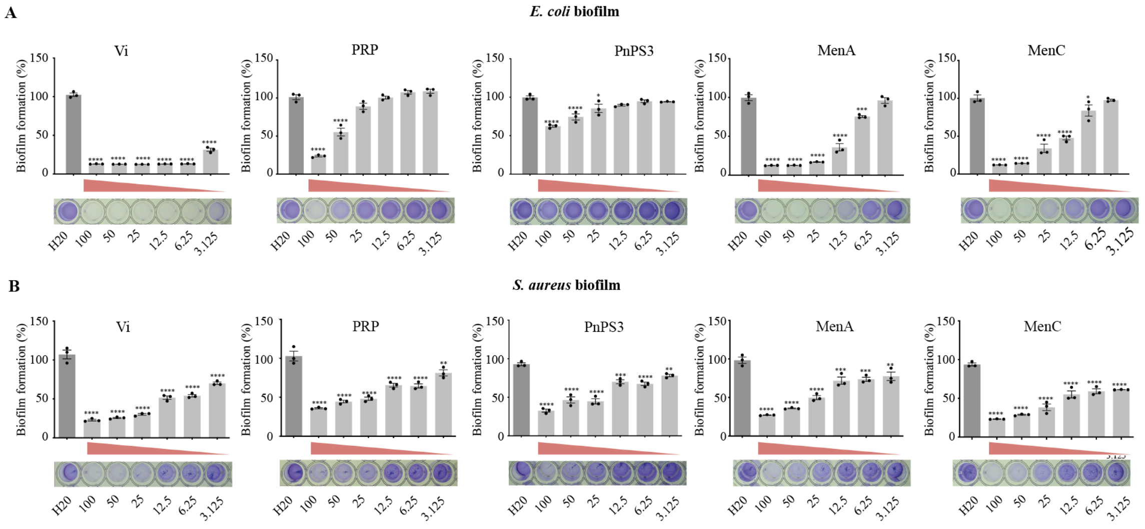

Supplementary Figure 2. **Level of antibiofilm activity of bacterial polysaccharides.** *E. coli* **(A)** and *S. aureus* **(B)** biofilm inhibition test (CV staining in microtiter plates) in presence of increasing concentrations of the indicated bacterial capsular polysaccharides produced by *Streptococcus pneumoniae*, *Salmonella enterica* serovar Typhi*, Haemophilus influenzae, Neisseria meningitidis.* Polysaccharide concentrations range from 3.125 to 100 µg/ml. Distilled water was used as a negative control. Each experiment corresponds to n=3 biologically independent experiments**.** Statistical analyses correspond to two-tailed unpaired *t-test* with Welch correction. * $p<0.05$; ** $p<0.01$; *** $p<0.001$; **** $p<0.0001$. Source data are provided as a Source Data file. Error bars represent SD.

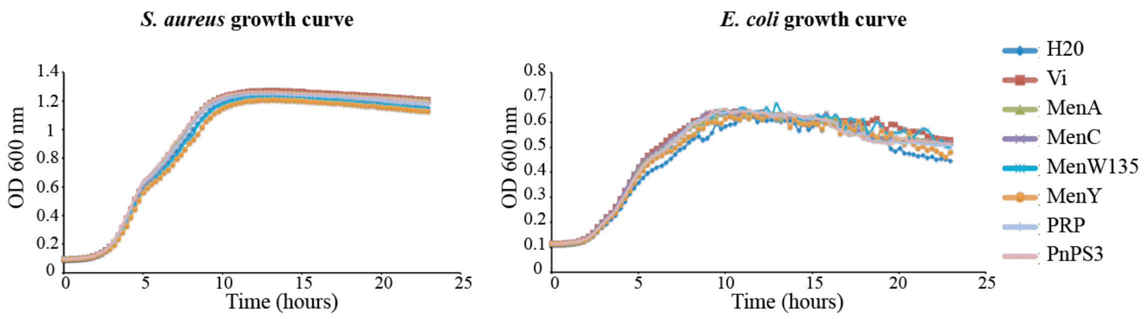

Supplementary Figure 3. **The identified antibiofilm polysaccharides are non-biocidal.** Growth curves of *S. aureus* and *E. coli* exposed to 100 µg/ml of each indicated tested macromolecule. The bacterial strains were inoculated in microtiter plates at OD_{600nm} of 0.05 and let to grow with agitation for 24 h at 37ºC. Bacterial growth was determined using a TECAN plate reader. Source data are provided as a Source Data file.

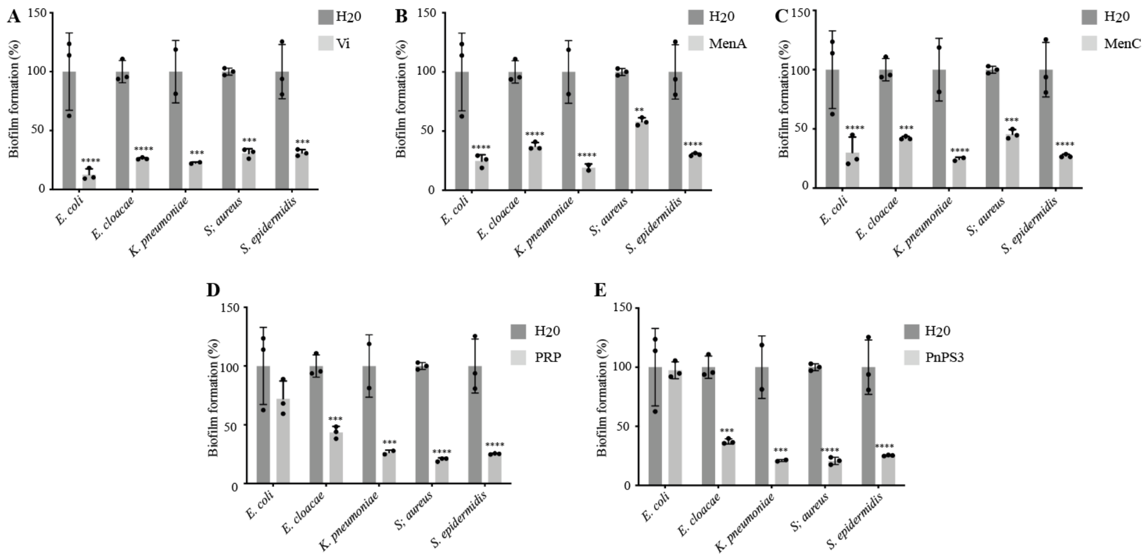

Supplementary Figure 4. **Spectrum of activity of 5 active antibiofilm polysaccharides.** Antibiofilm activity of purified polysaccharides Vi, MenA, PRP PnPS3 and MenC was assessed over a panel of relevant biofilm-forming pathogenic bacteria. Tested Gram-positive bacteria include *Staphylococcus aureus* 15981 and *Staphylococcus epidermidis* 0-47. Tested Gram-negative bacteria include *Escherichia coli*, *Enterobacter cloacae* 1092 and *Klebsiella pneumoniae* 21. Biofilm inhibition tests were performed in presence of 50 µg/ml of polysaccharide. Test of Vi (**A**), MenA (**B**), MenC (**C**) PRP (**D**), PnPS3 (**E**). Test of MenC activity was performed in a different experimental session. Distilled water was used as a negative control. Each experiment corresponds to n=3 biologically independent experiments**.** Source data are provided as a Source Data file. Statistical analyses correspond to two-tailed unpaired *t-test* with Welch correction. ** p<0.01; *** p<0.001. **** p<0.0001. Error bars represent SD.

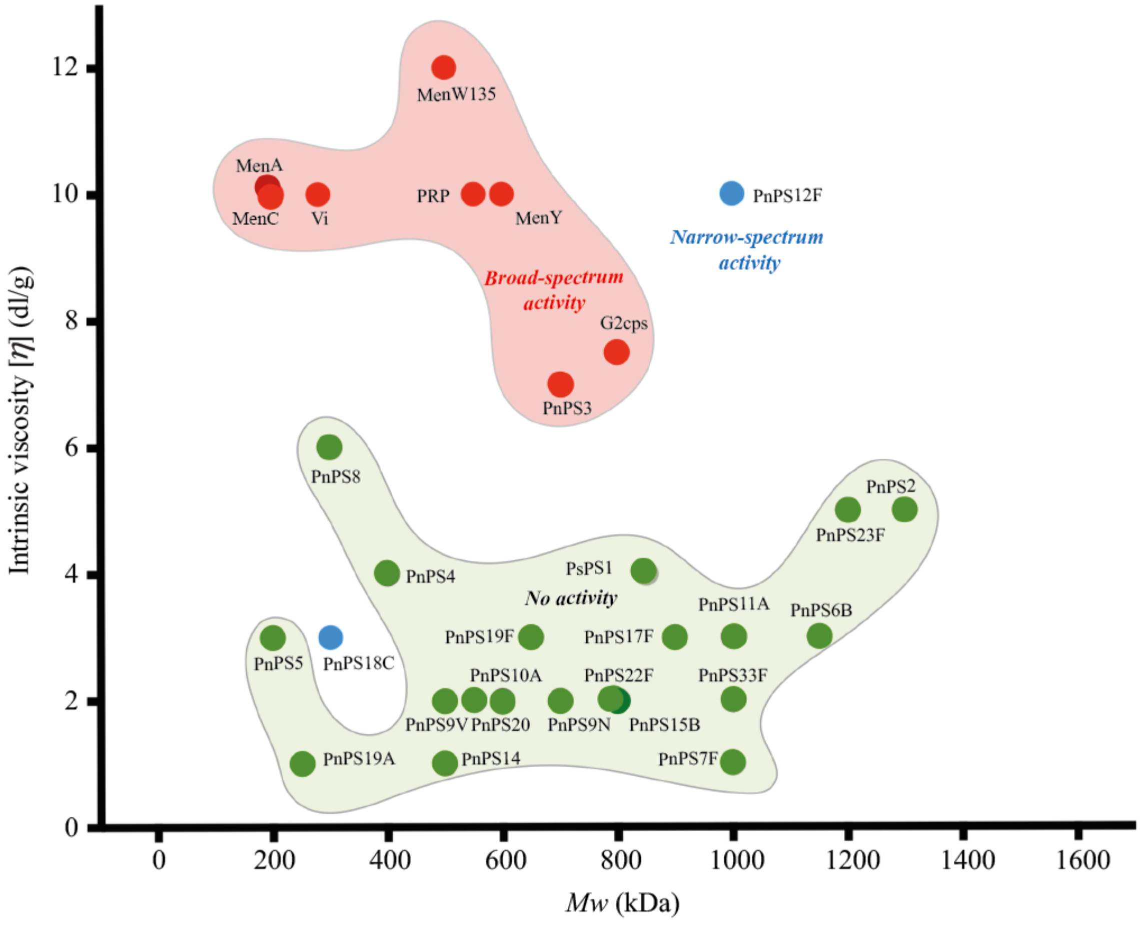

Supplementary Figure 5. **Classification of the tested polysaccharides according to their molecular weight (*Mw*) and intrinsic viscosity [η] as indicated in Table 1.** In red shading and red points: broad spectrum active macromolecules. In green shading and green points: inactive macromolecules. In blue points: polysaccharides with narrow spectrum antibiofilm activity.

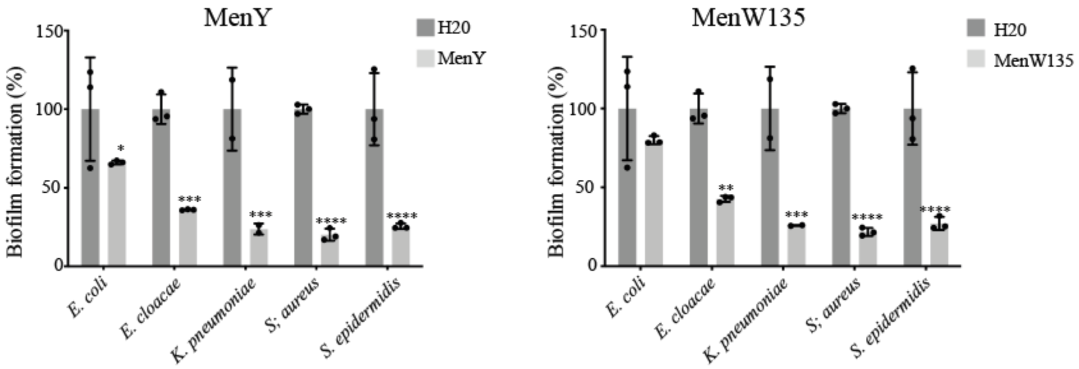

Supplementary Figure 6. **Spectrum of action of MenY and MenW135 over a panel of biofilm-forming pathogenic bacteria**. Tested Gram+ bacteria include *Staphylococcus aureus* 15981 and *Staphylococcus epidermidis* 047. Tested Gram- bacteria include *Escherichia coli* K12 MG1655 carrying the F plasmid, *Enterobacter cloacae* 1092 and *Klebsiella pneumoniae* 21. Biofilm inhibition tests were performed in the presence of 50 µg/ml of polysaccharide. Distilled water was used as a negative control. Each experiment corresponds to n=3 biologically independent experiments**.** Source data are provided as a Source Data file.
Statistical analyses correspond to two-tailed unpaired *t-test* with Welch correction. * $p<0.05$; ** $p<0.01$; *** $p<0.001$; **** $p<0.0001$. Error bars represent SD.

A

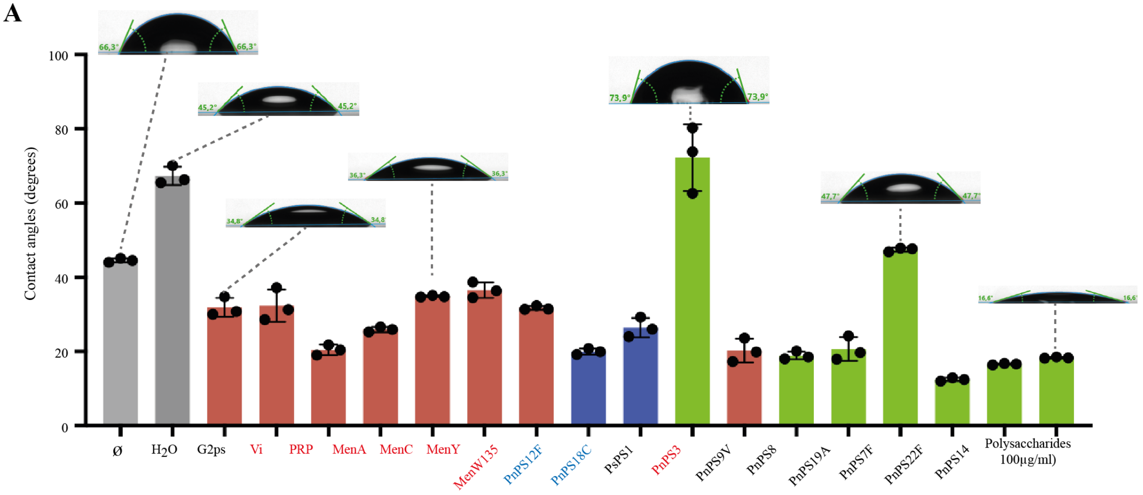

B

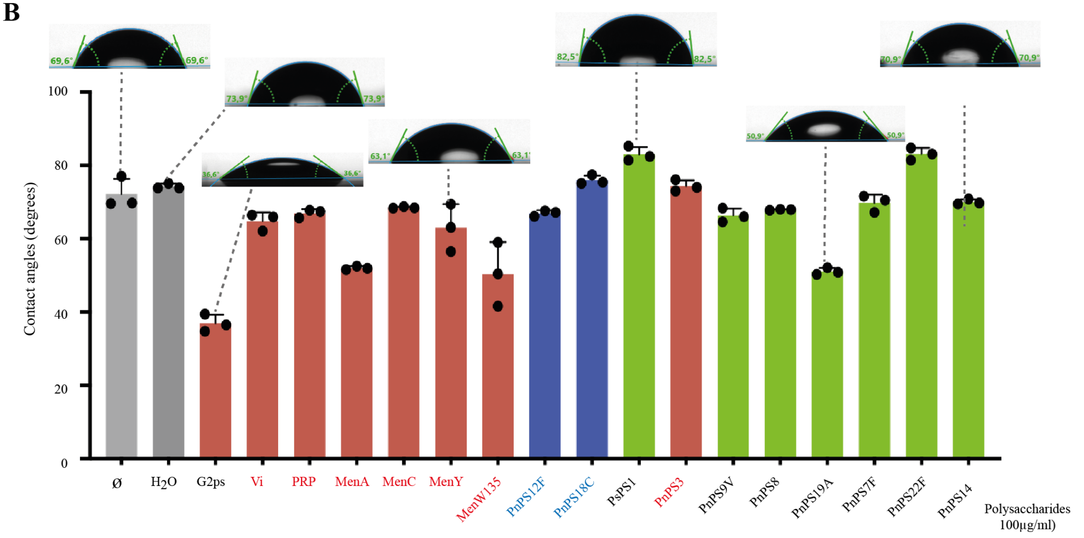

Supplementary Figure 7. **Determination of surface contact angle of a drop of water on glass or plastic surfaces treated with active and inactive polysaccharides**. Glass (**A**) and polyester plastic (**B)** microscopy slides untreated, treated with H_2O or treated with active broad-spectrum activity polysaccharides (in red), active narrow-spectrum activity polysaccharides (in blue) and inactive polysaccharides (in green) (see Table 1) in the presence of 100 µg/ml of polysaccharide. Representative pictures of water droplets used to determine contact angle are presented. Each experiment corresponds to n=3 biologically independent experiments**.** Source data are provided as a Source Data file. Statistical analyses correspond to two-tailed unpaired *t-test* with Welch correction. Error bars represent SD.

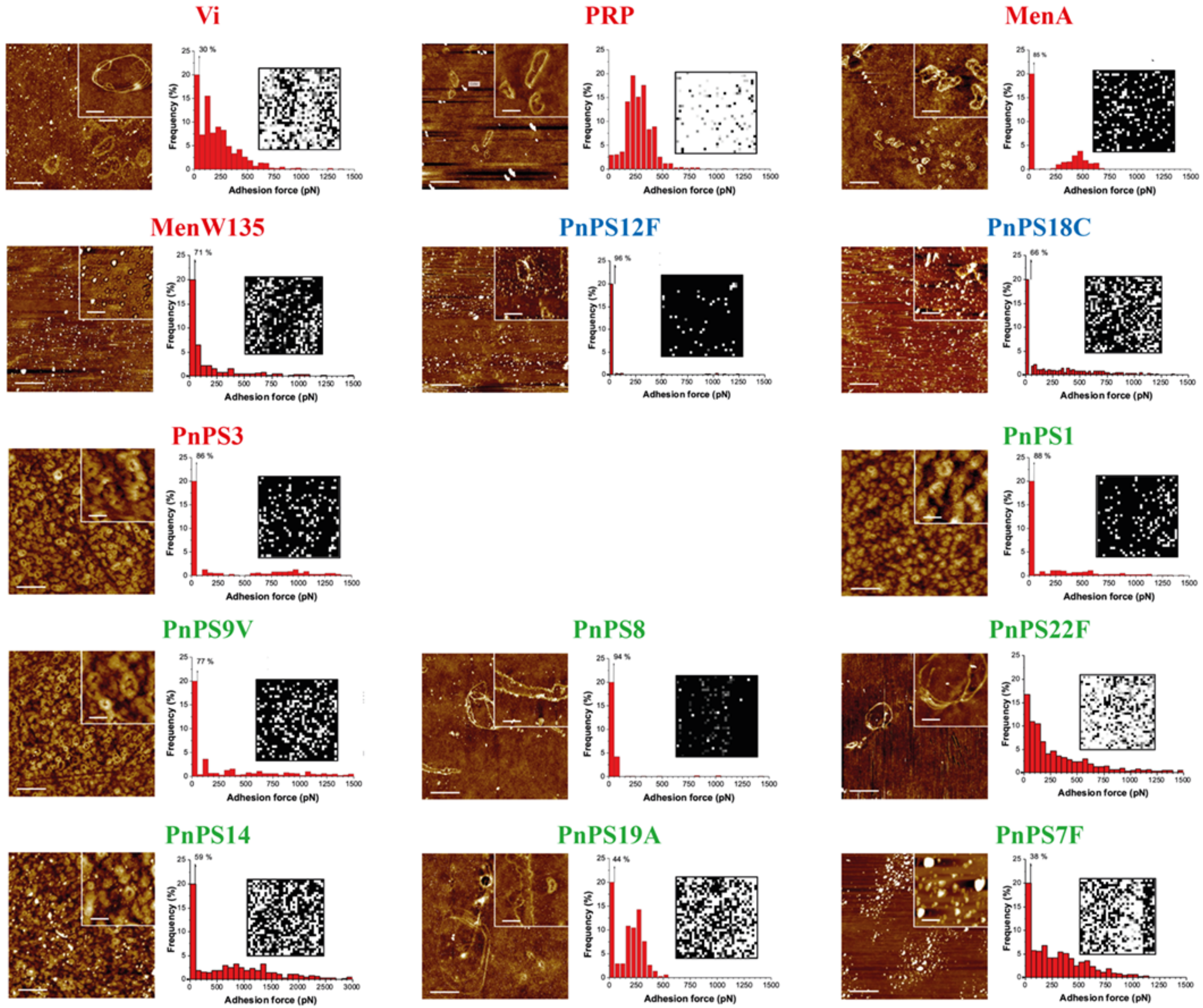
Vi
30 %
PRP
MenA
85 %
MenW135
71 %
PnPS12F
96 %
PnPS18C
66 %
PnPS3
86 %
PnPS1
88 %
PnPS9V
77 %
PnPS8
94 %
PnPS22F
PnPS14
59 %
PnPS19A
44 %
PnPS7F
38 %
Frequency (%)
Adhesion force (pN)

1

Supplementary Figure 8. **Representative AFM peak force images and adhesion force histograms of selected active and inactive polysaccharides.** Each couple of panels correspond to a given polysaccharide (specified). In each couple of panels, AFM images are on the left and adhesion force histograms on the right. Polysaccharides with broad-spectrum activity (indicated in red), narrow-spectrum activity polysaccharides (indicated in blue) and inactive polysaccharides (indicated in green) were deposited on a glass surface at 100 µg/mL and imaged in ultrapure water. Scale bars correspond to 1 µm. In each image, the inset shows the sample at higher magnification (scale bars = 250 nm). Adhesion force histograms (n=1024 force-distance curves) and corresponding adhesion force maps were recorded between AFM hydrophobic CH_3-tips and glass surfaces coated with selected active and inactive polysaccharides. Each map has been recorded on a 5 µm x 5 µm area. Grey scale: 150 pN, black pixels correspond to non-adhesive events.

SUPPLEMENTARY REFERENCES

1 Stroop, C. J., Xu, Q., Retzlaff, M., Abeygunawardana, C. & Bush, C. A. Structural analysis and chemical depolymerization of the capsular polysaccharide of Streptococcus pneumoniae type 1. *Carbohydr Res* **337**, 335-344 (2002).

2 Geno, K. A. *et al.* Pneumococcal Capsules and Their Types: Past, Present, and Future. *Clin Microbiol Rev* **28**, 871-899, doi:10.1128/cmr.00024-15 (2015).

3 Jansson, P. E., Lindberg, B., Anderson, M., Lindquist, U. & Henrichsen, J. Structural studies of the capsular polysaccharide from Streptococcus pneumoniae type 2, a reinvestigation. *Carbohydr Res* **182**, 111-117 (1988).

4 Reeves, R. E. & Goebel, W. F. Chemoimmunological studies on the soluble specific substance of pneumococcus: the structure of the type III polysaccharide. *J Biol Chem* **139**, 511-519 (1941).

5 Jones, C., Currie, F. & Forster, M. J. N.m.r. and conformational analysis of the capsular polysaccharide from Streptococcus pneumoniae type 4. *Carbohydr Res* **221**, 95-121 (1991).

6 Talaga, P., Bellamy, L. & Moreau, M. Quantitative determination of C-polysaccharide in Streptococcus pneumoniae capsular polysaccharides by use of high-performance anion-exchange chromatography with pulsed amperometric detection. *Vaccine* **19**, 2987-2994, doi:10.1016/s0264-410x(00)00535-1 (2001).

7 Jansson, P. E., Lindberg, B. & Lindquist, U. Structural studies of the capsular polysaccharide from Streptococcus pneumoniae type 5. *Carbohydr Res* **140**, 101-110 (1985).

8 Kenne, L., Lindberg, B. & Madden, J. K. Structural studies of the capsular antigen from Streptococcus pneumoniae type 26. *Carbohydr Res* **73**, 175-182 (1979).

9 Moreau, M., Richards, J. C., Perry, M. B. & Kniskern, P. J. Application of high-resolution n.m.r. spectroscopy to the elucidation of the structure of the specific capsular polysaccharide of Streptococcus pneumoniae type 7F. *Carbohydr Res* **182**, 79-99 (1988).

10 Jones, J. K. & Perry, M. B. The Structure of the Type VIII Pneumococcus specific po1ysaccharide. *J Am Chem Soc* **79**, 2787-2793 (1956).

11 Rutherford, T. J., Jones, C., Davies, D. B. & Elliott, A. C. NMR assignment and conformational analysis of the antigenic capsular polysaccharide from Streptococcus pneumoniae type 9N in aqueous solution. *Carbohydr Res* **265**, 79-96 (1994).

12 Calix, J. J. *et al.* Biochemical, genetic, and serological characterization of two capsule subtypes among Streptococcus pneumoniae Serotype 20 strains: discovery of a new pneumococcal serotype. *J Biol Chem* **287**, 27885-27894, doi:10.1074/jbc.M112.380451 (2012).

13 Jones, C. Full assignment of the proton and carbo NMR spectrum of the capsular polysaccharide from Streptococcus pneumoniae serotype 10A. *Carbohydr Res* **269**, 175-181 (1995).

14 Calix, J. J., Nahm, M. H. & Zartler, E. R. Elucidation of structural and antigenic properties of pneumococcal serotype 11A, 11B, 11C, and 11F polysaccharide capsules. *J Bacteriol* **193**, 5271-5278, doi:10.1128/JB.05034-11 (2011).

15 Leontein, K., Lindberg, B. & Lonngren, J. Structural studies of the capsular polysaccharide from Streptococcus pneumonia type 12F. *Can J Chem* **59**, 2080-2085 (1980).

16 Lindberg, B., Lonngren, J. & Powell, D. A. Structural studies on the specific type-14 pneumococcal polysaccharide. *Carbohydr Res* **58**, 177-186 (1977).

17 Jones, C. & Lemercinier, X. Full NMR assignment and revised structure for the capsular polysaccharide from Streptococcus pneumoniae type 15B. *Carbohydr Res* **340**, 403-409, doi:10.1016/j.carres.2004.12.009 (2005).
18 Jones, C., Whitley, C. & Lemercinier, X. Full assignment of the proton and carbon NMR spectra and revised structure for the capsular polysaccharide from Streptococcus pneumoniae type 17F. *Carbohydr Res* **325**, 192-201 (2000).
19 Lugowski, C. & Jennings, H. J. Structural determination of the capsular polysaccharide of Streptococcus pneumoniae type 18C (56). *Carbohydr Res* **131**, 119-129 (1984).
20 Katzenellenbogen, E. & Jennings, H. J. Structural determination of the capsular polysaccharide of Streptococcus pneumoniae type 19A (57). *Carbohydr Res* **124**, 235-245 (1983).
21 Jennings, H. J., Rosell, K. G. & Carlo, D. J. Structural determination of the capsular polysaccharide of Streptococcus pneumonia type-19 (19F). *Can J Chem* **58**, 1069-1074 (1980).
22 Richards, J. C., Perry, M. B. & Kniskern, P. J. Structural analysis of the specific capsular polysaccharide of Streptococcus pneumonia type 22F. *Can J Chem* **67**, 1038-1050 (1989).
23 Richards, J. C., Perry, M. B. & Carlo, D. J. The specific capsular polysaccharide of Streptococcus pneumoniae type 20. *Can J Biochem Cell Biol* **61**, 178-190 (1983).
24 Lemercinier, X. & Jones, C. Full assignment of the 1H and 13C spectra and revision of the O-acetylation site of the capsular polysaccharide of Streptococcus pneumoniae Type 33F, a component of the current pneumococcal polysaccharide vaccine. *Carbohydr Res* **341**, 68-74, doi:10.1016/j.carres.2005.10.014 (2006).
25 Skovsted, I. C. *et al.* Purification and structure characterization of the active component in the pneumococcal 22F polysaccharide capsule used for adsorption in pneumococcal enzyme-linked immunosorbent assays. *Vaccine* **25**, 6490-6500, doi:10.1016/j.vaccine.2007.06.034 (2007).
26 Heyns, K. & Kiessling, G. Strufturaufklarung des Vi-Antigens aus Citrobacter freundii (E. coli) 5396/38. *Carbohydr Res* **3**, 340–353. (1967).
27 Crisel, R. M., Baker, R. S. & Dorman, D. E. Capsular polymer of Haemophilus influenzae, type b. I. Structural characterization of the capsular polymer of strain Eagan. *J Biol Chem* **250**, 4926-4930 (1975).
28 Lemercinier, X. & Jones, C. Full 1H NMR assignment and detailed O-acetylation patterns of capsular polysaccharides from Neisseria meningitidis used in vaccine production. *Carbohydr Res* **296**, 83-96 (1996).
29 Valle, J. *et al.* Broad-spectrum biofilm inhibition by a secreted bacterial polysaccharide. *Proc Natl Acad Sci U S A* **103**, 12558-12563 (2006).
30 Mobley, H. L. *et al.* Pyelonephritogenic Escherichia coli and killing of cultured human renal proximal tubular epithelial cells: role of hemolysin in some strains. *Infect Immun* **58**, 1281-1289 (1990).
31 Rendueles, O. *et al.* Screening of Escherichia coli species biodiversity reveals new biofilm-associated antiadhesion polysaccharides. *mBio* **2**, e00043-00011, doi:10.1128/mBio.00043-11 (2011).
32 Valle, J. *et al.* SarA and not sigmaB is essential for biofilm development by Staphylococcus aureus. *Mol Microbiol* **48**, 1075-1087 (2003).
33 Heilmann, C., Gerke, C., Perdreau-Remington, F. & Gotz, F. Characterization of Tn917 insertion mutants of Staphylococcus epidermidis affected in biofilm formation. *Infect Immun* **64**, 277-282. (1996).
